# The Role of Ancient Grains in Alleviating Hunger and Malnutrition

**DOI:** 10.3390/foods12112213

**Published:** 2023-05-31

**Authors:** Mahsa Majzoobi, Shima Jafarzadeh, Shahla Teimouri, Mehran Ghasemlou, Milad Hadidi, Charles S. Brennan

**Affiliations:** 1 Biosciences and Food Technology, RMIT University, Bundoora West Campus, Plenty Road, Melbourne, VIC 3083, Australia; shahla.teimouri2@rmit.edu.au (S.T.); mehran.ghasemlou2@rmit.edu.au (M.G.); charles.brennan@rmit.edu.au (C.S.B.); 2 School of Civil and Mechanical Engineering, Curtin University, Bentley, WA 6102, Australia; s.jafarzadehkhosravi@ecu.edu.au; 3 Department of Organic Chemistry, Faculty of Chemical Sciences and Technologies, University of Castilla-La Mancha, 13001 Ciudad Real, Spain; milad.hadidi@gmail.com

**Keywords:** sustainable grains, combating hunger, malnutrition, ancient cereals

## Abstract

Meeting the United Nation’s sustainable development goals for zero hunger becomes increasingly challenging with respect to climate change and political and economic challenges. An effective strategy to alleviate hunger and its severe implications is to produce affordable, nutrient-dense, and sustainable food products. Ancient grains were long-forgotten due to the dominance of modern grains, but recently, they have been rediscovered as highly nutritious, healthy and resilient grains for solving the nutrition demand and food supply chain problems. This review article aims to critically examine the progress in this emerging field and discusses the potential roles of ancient grains in the fight against hunger. We provide a comparative analysis of different ancient grains with their modern varieties in terms of their physicochemical properties, nutritional profiles, health benefits and sustainability. A future perspective is then introduced to highlight the existing challenges of using ancient grains to help eradicate world hunger. This review is expected to guide decision-makers across different disciplines, such as food, nutrition and agronomy, and policymakers in taking sustainable actions against malnutrition and hunger.

## 1. Introduction

Hunger is a major problem in developing countries, and it is mostly related to food shortages/famine caused by various factors, including environmental stresses, geo–economic and political issues. However, in developed countries where food is abundant, hidden hunger or malnutrition caused by an imbalanced intake of nutrients is often observed. Despite the fact that one of the Sustainable Development Goals (SDG, Goal 2) of the UN is to eradicate hunger by 2030, the incidences of hunger and food insecurity are increasing. Recent reports by the UN show that the number of people affected by hunger has increased rapidly over the last five years and has reached about 828 million people in 2021, with a prediction that it will affect 670 million people in 2030. In 2021, nearly 2.3 billion people (mostly women and children) were severely or moderately food insecure, and about 3.1 billion people could not afford a healthy diet [1]. With the increasing incidents of climate shocks, geopolitical issues and disruption of the food supply chain, achieving the UN goal of zero hunger has become more critical and also more challenging [2].

Among different food sources, cereals have great roles in tackling hunger since they are the main staple food around the world and have the greatest shares in providing energy and other vital nutrients for humans [3]. Thus, one of the strategies to achieve zero hunger is to maintain the genetic diversity of the grains and produce nutrient-dense grains with improved health benefits that are highly resistant to environmental stresses and diseases and also can be readily processed into quality foods (Goal 2: Zero Hunger—United Nations Sustainable Development).

In terms of genetic background, cereals are dived into “modern” and “ancient” cereals. Unlike modern grains, ancient grains are under-utilised crops that have not been selected by breeding programs [4]. Until now, many aspects of ancient grains have been discovered, including their nutrient and health benefits, physicochemical properties, food applications and their contribution to food sustainability and diversity [5,6,7,8]. Thus, it is critical to find more applications for ancient cereals, especially to address global food challenges.

The main aim of this review article is to discuss the opportunities and challenges of ancient cereals as versatile crops to address the UN SDG of zero hunger as well as the global issue of malnutrition. This review paper has collected previous knowledge published on all types of ancient cereals in terms of physicochemical properties, nutritional profile and food industry applications.

This collective information can be of interest to researchers, grain breeders, producers, food manufacturers, climate advocates and policymakers to obtain a better understanding of how ancient grains can diversify our foods, especially as a solution for global hunger. It can also assist the food industry in making informed decisions and include more ancient grains in food products to produce healthier and more sustainable foods.

## 2. Ancient Cereals

Ancient cereals are those species of grains that have not been subjected to any selection or breeding by humans and have maintained specific genetic properties from their wild ancestors, such as ear height, low harvest index, brittle rachis and brittle individual variation [4]. Ancient grains include varieties of wheat (Spelt, Khorasan wheat or Kamut, Einkorn and Emmer); green wheat, barley; wild rice, oats; sorghum; millets, and pseudocereals of teff, amaranth; buckwheat and quinoa. In some references, freekeh and bulgur have been considered ancient grains even though they are made from ordinary wheat [5].

Many ancient grains are ancestors of modern grains. For example, the crossing between a diploid species of chamois (*Aegilops tauschii* Coss.) and Emmer (ancient wheat) resulted in Spelt, which was mutated over several generations to convert into common wheat [6].

At the dawn of civilisation, ancient cereals used to provide a vital food source in the human diet. However, over the centuries, the selection of domesticated species with higher production yields and improved techno-functional properties has led to a dramatic decline in the production of other grains and the dominance of only a few grains—known as leading cereals—including wheat, rice, corn and barley [7]. This has generated significant food security concerns, especially with increasing the adverse impacts of climate change and supply chain disruptions due to the global pandemic and geopolitical and socioeconomic issues [8]. However, in recent years, ancient grains are regaining worldwide attention for a variety of reasons. The production of ancient grains is regarded as being environmentally friendly, generating low carbon footprints as they require less irrigation, pesticides and fertilisers compared to many normal grains. Ancient grains are also suitable for climate-smart agriculture since they can tolerate harsh growing conditions [9]. In addition, ancient grains are recognised as rich sources of nutrients and bioactive compounds with numerous health benefits [4]. Therefore, they are a key player in developing sustainable food systems and are well-positioned to tackle food insecurity caused by the ongoing climate change.

## 3. Ancient Grains vs. Modern Grains

The comparison between ancient and modern grains, especially in terms of composition and nutritional value, is still controversial and needs further accurate research. This is mostly because of the lack of comprehensive studies, as the full impacts of plant genetics (g), environmental factors € and their interactions (g × e) on the physicochemical properties of the grains have not been fully considered and determined in many studies, which hinders the accuracy of the findings [10]. However, from some previous studies that factored in the variables affecting the physicochemical properties of the grains, it is obvious that ancient grains have lower yields than modern grains, which is one of their main limitations. Nevertheless, increasing the yield of the modern grains diminishes their protein contents and other valuable nutrients that can negatively affect the health benefits and technological properties of the grains, such as quality of bread making [6]. Ancient grains are more tolerant to biotic and abiotic stresses [8] and often contain more protein, dietary fibre, bioactive compounds and antioxidant activity and show improved health benefits (see Table 1 and Table 2).

Comparing ancient wheat and modern wheat for their potential to elicit coeliac disease has found similar immunoreactivity of both cultivars and, hence, the breeding of modern wheat is not responsible for the prevalence of coeliac disease [45,46].

Preliminary in vivo and in vitro studies indicated that the consumption of different ancient grains could be better tolerated by non-coeliac wheat-sensitive individuals and those who suffer from irritable bowel syndrome. However, children aged 3–13 years old with wheat sensitivity seem to show similar reactions to both ancient and modern wheat cultivars.

A few individuals only sensitised to alpha-amylase/trypsin inhibitor showed no reaction to Einkorn since the corresponding gene is missing in this grain [47]. For wheat grains, it has been reported that the starch digestibility of bread made with ancient wheat and modern wheat is not related to the release year of the cultivar and indicated it is doubtful that the wheat breeding program has affected starch digestibility [48].

Ancient wheat and barley are considered gluten-containing grains and are unsafe for coeliac patients. Although ancient wheat has higher gluten than modern wheat, their gliadins are in the form of more digestible and less toxic but still unsafe for coeliac patients [18,49].

## 4. Physicochemical, Nutritional Profile and Health Benefits of the Ancient Grains

### 4.1. Wheat

Archaeological evidence shows that wheat most likely appeared first in Lebanon, Syria, Turkey, Egypt and Ethiopia. The domestication of wheat is likely to have begun around 10,000 years ago in the Fertile Crescent, and since then, wheat has been regarded as the most cultivated crop in the world [25]. The most common ancient wheat species include Einkorn (*Triticum monococcum*), Emmer (*Triticum dicoccum*), Khorasan (*Triticum turgidum* ssp. *turanicum*) and Spelt (*Triticum spelta*). Wheat grains have lengths mainly between 5 and 9 mm and shapes that may vary from spherical to flattened. The 1000-kernel weight of Spelt is ~44 g, which is much higher than that of Einkorn (~28 g) [50]. As shown in Table 1, Einkorn and Emmer wheat are typically composed of 53–72% carbohydrates (mainly starch), 12.5–12.7% protein, 10.6–12.5% dietary fibre, 2.1% lipids and 1–3% minerals. The main interest in the worldwide adaptation of ancient wheat species could be related to their high protein contents and production yield and their high tolerance to many biotic and abiotic stresses. The high-yielding modern wheat produced by breeding programs often have lower protein content than ancient grains. Higher protein contents (~18%) in Einkorn wheat than other cultivars of Emmer (~15%) and Spelt (~13%) have been reported [6]. Ancient wheat species contain slightly lower carbohydrate contents than modern wheat. Within the ancient wheat group, Spelt and Einkorn have the lowest carbohydrate contents (~67–69%). The starch content of ancient wheat species is often lower than modern cultivars, and its composition varies greatly from modern wheat. For instance, Einkorn has lower resistant starch content (25.6 g/kg) than modern wheat (30–88 g/kg), whereas Spelt, Emmer and Einkorn contain 30–32% rapidly digestible starch, 26–59% slowly digestible starch and 2.3–2.4% resistant starch [51]. Einkorn showed a higher content of lipids compared to common wheat. Modern wheat varieties may have a rich content of mineral and dietary fibre compared to Einkorn and Emmer wheat. Ancient wheat species also contain fewer anti-nutrients than common wheat. The phytic acid contents of the Einkorn and Emmer wheat were between 1594 and 1863 mg/100 g [52].

A comparison between old and modern wheat cultivars showed higher health-relevant benefits of old cultivars. It has been indicated that the consumption of bakery products made with Khorasan wheat can enhance the immune functions in patients with severe symptoms and sleep disorders [6].

### 4.2. Green Wheat (Freekeh)

Premature green wheat, or freekeh, is an ancient whole grain with a history spanning thousands of years. Green wheat is produced from wheat harvested early, at the end of the milky stage, when culms and spikes are green. Grain shape, plumpness and greenness determine the quality of freekeh. Green wheat has a high initial moisture content that varies from 40–45% (wet basis), but during the drying process, it loses about 40% of its weight [30,53,54]. Depending on moisture content, kernel length, width and thickness differ from 6.24 to 6.66 mm, 3.65 to 4.22 mm and 3.43 to 3.85 mm, respectively. In addition, the mass of 1000 seeds varies from 15 to 51 g at different maturation stages [55]. To produce green wheat, often, immature durum wheat (*Triticum durum*) and, sometimes, immature bread wheat (*Triticum aeisvum*) are used. The *Zenit* and *Diyarbakır* spp. durum wheat is favoured for this purpose [30,56].

Green wheat is used as a raw material in the production of many foods and healthy drinks. Roasted green wheat, which is commonly known as freekah (also known as frekeh or frikah), has been a popular staple food in Middle Eastern, North African, and Chinese cuisines for centuries. Roasting improves the flavour of the grains; however, it causes huge losses to their nutritional quality [30].

Green wheat contains 73–80% carbohydrates, 11–15% protein and 12–19% dietary fibre (Table 1). The starch content of green wheat is 45% and 68%, and its resistant starch content is about 8.0 to 10%. Due to its higher resistant starch and dietary fibre content and lower GI (52–54) compared to wheat, green wheat is more suitable for people with diabetes and for weight control.

Green wheat has a significantly greater proportion of essential amino acids, particularly lysine, methionine and threonine, and has better protein digestibility than normal wheat. Its total fatty-acid content varies from 1.32 to 2.7%, which is higher than that of yellow wheat. Palmitic acid is the dominant saturated fatty acid, and linoleic acid is the dominant unsaturated fatty acid [56].

The total mineral content in green wheat is higher than that in mature yellow wheat. Green wheat is a rich source of bioactive compounds. The total phenolic content, flavonoid content and antioxidant properties of green wheat are about twice that of wheat. Nevertheless, green wheat contains antinutrient compounds, such as phytate (660–700 mg/100 g), which is formed during the maturation of the seeds [30,56]. The freekeh grains harvested at earlier stages have the lowest phytic acid and phytate contents, which are nutritionally quite desirable [55]. The food applications of freekeh are limited to some traditional and homemade foods; however, due to increased knowledge about the nutritional and health benefits of green wheat, an increase in the global consumption of green wheat is expected. A few studies have shown the applications of green wheat in the formulation of healthy foods. For example, it has been found that the inclusion of green-wheat flour in the preparation of noodles can enhance the quality of the noodles and reduce their predicted GI [30].

### 4.3. Barley

Barley is a highly nutritious and adaptable ancient grain crop with growing cultivation all over the world. It is globally cultivated as the fourth most popular cereal in terms of production after wheat, rice and corn. Barley may have originated in Southeast Asia, including China, Tibet and Nepal [57]. There is limited information on the domestication of barley grains. A study reported the genome sequences of ancient barley grains excavated at Yoram Cave in the Judean Desert in Israel [58]. This report suggested that barley grains cultivated in the present day closely resemble those of old cultivars, although there is evidence for gene flow between the two populations. Barley grains are generally larger than wheat, with a 1000-kernel weight of about 40–45 g, and appear with a bright, light-yellow colour. Typical barley cultivars have distinct two-layered cells with adherent hulls departed at harvest maturity. However, hull-less varieties of barley have a low prevalence but are cultivated from certain seeds [59]. Today, more than 70% of barley grains are used for animal feed, about 20% are used for malting and brewing industries, and only a very small fraction is directly used in the human diet [57].

The chemical composition and nutritional profile of barley are given in Table 1 and Table 2, respectively. Generally, barley contains protein (10–17%), carbohydrates (~65–68%), lipids (2–4%), dietary fibres (18–22%), β-glucan (4–9%), minerals (1.5–2.5%) and vitamins (~2%). It contains ~14–20% rapidly digestible starch, ~20–25% slowly digestible starch and about 2.2% resistant starch that would help regulate the rate of glucose release in barley-containing foods in the human body. Barley kernel contains several bioactive compounds, including β-glucans, lignans, phytosterols and polyphenols. The relatively high β-glucan content present in barley helps to lower serum cholesterol levels and control blood glucose and insulin resistance. Barley is also a good potential source of a range of vitamins, including B1 (0.35 mg/100 g), B2 (0.091 mg/100 g) and E (0.85–3.15 mg/100 g). More recently, research has focused on the nutritional profiles of germinated barley grains as a food ingredient that could be rich in antioxidant compounds useful in functional food applications [60].

### 4.4. Barley

Barley is a highly nutritious and adaptable ancient grain crop with growing cultivation all over the world. It is globally cultivated as the fourth most popular cereal in terms of production after wheat, rice, and corn. Barley may have been originated from South-Eastern Asia including China, Tibet and Nepal [55]. There is limited information on domestication of barley grains. A study reported the genome sequences of ancient barley grains excavated at Yoram Cave in the Judean Desert in Israel [56]. This report suggested that barley grains cultivated in present-day closely resemble those of old cultivars, although there is evidence for gene flow between the two populations. Barley grains are generally larger than wheat with a 1000 kernel weight of about 40–45 g and appear with a bright, light-yellow colour. Typical barley cultivars have distinct two-layered cell with adherent hulls departed at harvest maturity. However, hull-less varieties of barley have a low prevalence but are cultivated from certain seeds [57]. Today, more than 70% of barley grains are used for animal feed; about 20% are used for malting and brewing industries; and only a very small fraction is directly used in human diet [55].

The chemical composition and nutritional profile of barley are given in Table 1 and Table 2, respectively. Generally, barley contains protein (10–17%), carbohydrates (~65–68%), lipids (2–4%), dietary fibres (18–22%), β-glucan (4–9%), minerals (1.5–2.5%), and vitamins (~2%). It contains ~14–20% rapidly digestible starch, ~20–25% slowly digestible starch and about 2.2% resistant starch that would help regulate rate of glucose release barley containing foods in human body. Barley kernel contains several bioactive compounds including β-glucans, lignans, phytosterols and polyphenols. The relatively high β-glucan content present in barley, helps to lower serum cholesterol levels, control blood glucose and insulin resistance. Barley is also a good potential source for a range of vitamins including B1 (0.35 mg/100 g), B2 (0.091 mg/100 g), and E (0.85–3.15 mg/100 g). More recently research has focussed on the nutritional profiles of germinated barley grains as a food ingredient which could be rich in antioxidant compounds useful in functional food applications [58].

### 4.5. Oats

Oat (*Avena* L., *Poaceae* family) is a valuable cereal crop in many countries with a primary usage for animal feed, but due to its health benefits, its food applications are growing rapidly. However, the world production of oat for human food is still lower than other grains due to the lower yield and high cost of production and transport (due to the low density of oat grains) [30]. Oats have a 1000-kernel weight of about 34–35 g and have been grown from ancient times in many parts of the world, particularly in Northern and Eastern Europe [61].

As shown in Table 1, oats contain carbohydrates (75–80%), protein (10–15%), lipids (3–8%) and β-glucan (4%). In contrast to other cereals, a distinguished feature of oat grains is their high protein content and distinct and balanced amino acid composition. The amino acid composition of oat grains is superior to that of other cereals because its major storage protein is globulin, with higher concentrations of essential amino acids such as lysine than other cereals [62]. Oats are rich in carbohydrates, including ~60% starch with about 15% rapidly digestible starch, 8–9% slowly digestible starch and 76% resistant starch [63].

As shown in Table 2, oat is a highly nutritious crop and a rich source of soluble dietary fibre (β-glucan), functional and bioactive compounds, fatty acids (e.g., linoleic and oleic acids) and minerals, especially calcium, iron and zinc. These bioactive ingredients have been shown to enhance the antioxidant content of foods such as crackers or biscuits compared to wheat flour, thus indicating a potential use of oat flour as a nutritional enhancer for the food industry. The main nutritional implications and health benefits of oats in human diets are attributed to the presence of a significant amount of β-glucan that reduces blood cholesterol and glucose [63]. Phytic acids (270–290 mg/100 g) and tannins (38–46 mg/100 g) are the main antinutrients in oat [27].

### 4.6. Sorghum

Sorghum is a drought-tolerant cereal belonging to the *Poaceae* grass family and originating in the northeast quadrant of Africa. It is the world’s fifth most important cereal after wheat, rice, maize and barley, with over 58.7 million tons of total production in 2020. The United States is the most significant producer of this crop, followed by Nigeria, Ethiopia, India, Mexico and China [31,32]. Sorghum is a very genetically diverse crop, with over 24 diverse species identified to date. Notable among these is *S. bicolor*, known for its food use and considered one of the most important species in modern commercial breeding programs. *S. bicolor* originated from its wild progenitor *Sorghum bicolor* L. Moench subsp. *Verticilliflorum. Sorghum bicolor* (L.) Moench is categorised into five major races: *bicolor* (the primitive type), *guinea*, *caudatum*, *kafir* and *durra* with various physical and biochemical properties [27]. Sorghum varieties have been classified based on different characteristics. However, based on the end-use applications, sorghum is classified into five groups, including sweet sorghum (syrup and biofuel), grain (biofuel, human food and animal feed), fibre, forage/fodder (animal feed) and broomcorn (broom-making) [32].

Sorghum has small seeds with pigmented pericarp, and the most commercially available varieties are black, white and red [16,33]. White sorghum is used for food products, while red sorghum is utilised primarily in the alcohol distillation industry [34]. Sorghum grains are ovoid with one end more pointed; the grain diameter ranges between 4 and 8 mm, and the mean weight of 1000 grains varies from 20 to 60 g. As shown in Table 1, starch is the main component of sorghum (about 70%); however, sorghum grains show the highest content of resistant starch (4–21%) and lowest starch digestibility (~19–37% rapidly digestible starch) and glycemic index among cereal crops [35].

The major protein fractions in sorghum are prolamins (kafirins), followed by glutelins; however, it has a low content of essential amino acids such as lysine, methionine and isoleucine [36].

The lipid in sorghum grains is made up of saturated fats and a high concentration of unsaturated fatty acids. Sorghum, especially red sorghum, is a rich source of various phytochemicals, mainly phenolic acid (mostly ferulic acid), flavonoids and tannins, with substantial health-promoting effects (Table 2).

Sorghum grains, especially pigmented grains, have limited applications as human foods due to the presence of condensed tannins contributing to bitter taste, phytates, cyanogenic glycosides and trypsin inhibitors, which are considered the major antinutritional factors. However, varying food-processing methods such as sprouting, cooking, fermentation, steaming and flaking can reduce the antioxidants in sorghum [36]. In addition, low-tannin sorghum varieties have been identified and bred that have been used as an alternative for corn to feed animals [37]. It is also possible to reduce the tannin content of sorghum using food-processing methods such as milling followed by soaking in 0.3% Na_2_CO_3_ solution for 8 h [38]. Novel applications of sorghum include the production of plant-based protein, healthy foods and gluten-free products, and ethanol and biofuel production has emerged [39]. The digestibility of sorghum starch has been shown to vary dependent upon variety and may therefore be a useful flour-based ingredient for the optimisation of the glycaemic index of starch-based foods [40].

### 4.7. Millet

Millets are small-seeded species of cereal crops belonging to the family *Poaceae*, which originated in the arid and semi-arid regions of Asia and Africa. It has a short growing season and is resistant to pests and diseases. Millet has five genera: Panicum, Setaria, Echinochloa, Pennisetum and Paspalum [57]. The most important cultivated varieties of millets are foxtail millet (*Setaria italica*), pearl millet (*Pennisetum glaucum*), proso millet (*Panicum miliaceum*), barnyard millet (*Echinochola crusgalli*), finger millet (*Eleusine coracana*), brown top millet (*Panicum ramosum*), kodo millet (*Paspalum scrobiculatum*) and teff millet (*Eragrostis tef*). Millet is the sixth most high-yielding grain in the world, with a total annual production of 30.4 million tons, but is still considered an underutilised grain [31,57]. Millet seeds have small and round shapes with different colours. The seed size varies between 3 and 4 mm, the 1000-kernel weight of millet varieties is about 2.5–3.0 g and the bulk density and true density are about 0.67–0.55 gmL^−1^ and 1.36–1.79 gmL^−1^, respectively [57,64]. As shown in Table 1, the main constituent of millet is its starch (62 to 70%), and some reports indicated that millet contains about 4–5% resistant starch, 6–7% slowly digestible starch and ~10–11% rapidly digestible starch (RDS) [65]. The second major component of millet is protein. The amino acid profile of pearl millet is better than that of sorghum and maize, and is comparable to that of wheat, barley and rice, and lysine is the first limited amino acid in millet cultivars [66]. Among millets, finger millet is relatively better balanced in essential amino acids because it contains more lysine, threonine and valine. The crude fat content in finger millet has been reported in the range of 1.54 to 3.77%. Linolenic acid and oleic acid are the two dominant fatty acids in the millet varieties [67].

Among millets, finger millet is the richest source of calcium and iron, with levels higher than those of sorghum, barley, maize and wheat. Millet grains are rich in several phytochemicals, particularly phenolic compounds. Finger millet has been shown to have the highest phenolic content and antioxidant activities compared to proso and foxtail millets [67]. Millets also have antinutrients, such as phytic acid (296–620 mg/100 g), tannins (31–343 mg/100 g) and trypsin inhibitors, which may reduce the bioavailability of minerals [28]. Millets are often subjected to different processing methods such as dehulling, decortication, soaking, germination, malting, milling, cooking, roasting, popping, radiation and fermentation to improve the nutritional and sensory properties of millets for developing new food (Xiu et al., 2022). Millet has some food applications, including the production of gluten-free foods, bakery products and porridge [28,68].

### 4.8. Wild Rice

Wild rice, known as a health-promoting grain, is the seed of an aquatic plant belonging to the genus Zizania, family *Poaceae* [29]. Wild rice (*Zizania* spp.) originated from North America over 10,000 years ago and then dispersed into East Asia and other parts of the world [69]. It consists of four species: *Zizania palustris* L., *Zizania aquatica* L., *Zizania texana* H. and *Zizania latifolia* G [14].

The seeds of wild rice have long and narrow cylindrical shapes approximately 4.7 to 9.2 mm long and 1.6–2.8 mm wide. The grain colour of these wild rice varies from light red–brown to dark brown with a 1000-kernel weight of 23–37 g [69]. As shown in Table 1 and Table 2, wild rice is rich in minerals, vitamins, starch, dietary fibre, protein and antioxidant phytochemicals, and is low in fat. Wild rice contains about 56–79% starch as the main constituent. Wild rice starch has shorter chains of amylose and longer chains of amylopectin, which causes a slower in vitro digestion rate compared to that of domesticated rice. It contains about 60% rapidly digestible starch, ~4% slowly digestible starch and ~5% resistant starch [14,29]. The resistant starch content of the wild rice is about 10.8%, which is significantly higher than white rice (~1.4%) and red rice (~0.95%). It also contains about 6.8% dietary fibre content, which is considerably higher than that of red rice (~2.6%) and white rice (~0.42%) [70].

Protein (10–15.5%) is the second main constituent of wild rice, which is much higher in content and efficiency ratio than that in white rice (~10%) and red rice (~11%). The essential amino-acid profile of wild rice is generally better and more balanced than that of other grains. Threonine and lysine are the limiting amino acids in all varieties of wild rice [14,69].

As a whole grain, wild rice is a rich source of phenolic compounds and flavonoids, and this level of antioxidant phenolic compounds is 10–15 times higher than that of white rice. Ferulic acid is the predominant phenolic acid, followed by sinapic acid and p-coumaric acid. Other phytochemical constituents of wild rice are flavonoid glycosides and flavan-3-ols. In addition to phenolic compounds, anthocyanins and carotenoids such as lutein were found in wild rice, thus providing a more complete profile of the antioxidants in wild rice [14,69,70]. Traditionally, wild rice has been exploited to treat a variety of ailments in Chinese medicinal practice [29]. Several health benefits of wild rice are listed in Table 2.

### 4.9. Amaranth

Amaranth (*Amaranthus* spp.), a pseudocereal and a member of the *Amaranthaceae* family, is a less explored species with an excellent nutritional profile for human consumption. Amaranth has a diverse range of 60 species but has three common species (*Amaranth hypochodriacus, Amaranth cruentus* and *Amaranth caudatus*) domesticated for their seeds [71]. China is the largest producer of amaranth in the world, followed by the United States, Canada and Argentina. Owing to its high nutritional quality, such as balanced content of essential amino acids and unsaturated fatty acids, as well as being gluten-free, amaranth is gaining importance among consumers, food producers and the scientific community [72]. Amaranth protein contains a high amount of lysine, which is a limited amino acid in almost all cereals and other pseudocereal grains [73]. Its protein is also abundant in cysteine and methionine, two essential amino acids that contain sulphur. The *Amaranthus* species is recognised as a source of important vitamins, such as vitamin C, carotene, folate and B_6_, among cereals and vegetables (see Table 2). Aside from its nutritional value, amaranth grain includes several bioactive compounds with potential health benefits. The total phenolic content in amaranth grains ranges from 21.2 to 57.0 mg gallic acid/100 g dry weight, mainly containing ferulic acid followed by quercetin and isorhamnetin. Phytate (0.09%) and saponins (4.96 mg/100 g) are the main antinutrients in amaranth [23,74].

### 4.10. Quinoa

Quinoa (*Chenopodium quinoa* Willd.) is a pseudocereal commonly known as the “golden grain” and has long been considered a source of nourishment and sustenance for Andean indigenous societies. Quinoa grain is mainly cultivated in the South American Andes region; however, over the past decades, it has been introduced in North America, Europe, Africa and Australia. Quinoa production has continuously expanded over the last few decades, and by 2013, the international year of quinoa, quinoa production and consumption had increased dramatically [23,74].

Quinoa flour is used to make a variety of toasted and baked goods, including bread, cookies, biscuits, noodles, pasta and pancakes. In addition, quinoa grains can be fermented to produce alcoholic beverages such as beer owing to its high starch level. Owing to its high nutritional quality and adaptability, quinoa is traditionally used in livestock feeding. Quinoa grains contain no gluten. Additionally, it has a high amount of nutrient ingredients such as proteins, dietary fibres, vitamins, fatty acids and minerals (see Table 1 and Table 2 for chemical composition and nutritional profile). The protein content of quinoa grains varied from 12.8 to 16.7%, which is higher than those of corn, rice and barley. The two main storage proteins in quinoa grain are albumins (35%) and globulins (37%). Quinoa proteins are recognised as high-quality proteins due to their great amount and well-balanced composition of essential amino acids. Quinoa protein contains a high concentration of lysine (2.4–7.8 g/100 g protein), methionine (0.3–9.1 g/100 g protein) and threonine (2.1–8.9 g/100 g protein), which are the limiting amino acids in ancient cereals such as maize and wheat [23,74,75].

Similar to other grains, starch is the most important carbohydrate component (32–69% of total carbohydrates). Its total dietary fibre content (7.0–16.5%) is comparable to modern cereals such as wheat. In addition to having a high protein content and good bioavailability, quinoa also has an intriguing lipid content (3.9–7.4%) that is higher than that of wheat and rice, making it a viable oil seed alternative source. The vitamin content, such as for vitamin C, E and folic acid, are greater than those of most other grains, and there is great potential to use quinoa as a functional food ingredient in mainstay food-processing applications. Quinoa has several health benefits in high-risk groups such as children and the elderly, as well as having prebiotic and probiotic effects [41]. However, it also contains phytate, saponin, tannins and protease inhibitor as the main antinutrients [18].

### 4.11. Teff

Teff *(Eragrostis tef*) is a nutritious, gluten-free pseudocereal grain that is native to Ethiopia and Eritrea. It is a staple food in these countries and is often used to make traditional dishes such as injera (a sour fermented pancake-like flat bread). Teff is a rich source of protein (12–15%) and fibre (6–8%) [21]. Teff contains a high level of lysine, which is an essential amino acid that is important for growth and tissue repair. Teff is a good source of minerals, including iron and calcium, which are beneficial for individuals with anaemia or osteoporosis. Teff is also a good source of resistant starch, which can help improve digestion and blood sugar control [18,21].

Teff also contains a variety of phytochemicals and antioxidants, including phenolic acids and flavonoids, which have been shown to have anti-inflammatory and anti-cancer properties and can reduce the risk of chronic diseases [42].

Phytic acid, tannins and protease inhibitors are the main antinutritional factors in teff [21]. To minimise the negative effects of these compounds, traditional methods of processing, such as fermentation, soaking and germination, can be used to reduce the levels of anti-nutritional compounds in teff. Teff can be ground into flour and used to make a variety of baked goods, including bread, pancakes and cakes. It can also be cooked and eaten as a porridge or added to salads and stews. Phytate, tannins, oxalates and saponins are the main antinutrients in teff [21,76].

### 4.12. Chia

Chia (*Salvia hispanica*) is a pseudocereal native to Mexico and Central America. It is a member of the mint family (*Lamiaceae*) and is closely related to other species such as sage and oregano. The chia seeds are small and oval in shape, measuring about 1–2 mm in diameter. They are black, brown or white in colour and have a glossy surface. The chia plant is drought-tolerant, making it suitable for dryland farming [43,77,78]. Chia seeds are an excellent source of dietary fibre (~34%), lipids (~33%) and protein (~18%). The protein content in chia seeds is composed of essential amino acids, such as lysine and arginine, and non-essential amino acids, such as alanine and aspartic acid. The chia seed lipid is rich in polyunsaturated acids with beneficial health impacts and, recently, has been extracted and characterised for food applications. Chia seeds are great sources of bioactive compounds such as omega-3 fatty acids (60–64%) and are a good source of minerals. Phytate and trypsin inhibitors are the major antinutrients in chia seeds [22,43,77,78].

### 4.13. Buckwheat

Buckwheat (*Fagopyrum esculentum*) is a pseudocereal that belongs to the family *Polygonaceae*. It is small and dark-coloured, typically brown or black, and is often used as a grain-like food source. Buckwheat is a hardy plant that can grow in a variety of soil types and climates, it is tolerant to frost and can be grown as a cover crop or as a green manure crop [44,74]. The seed of the buckwheat plant is a good source of carbohydrates (~65%), mainly in the form of complex carbohydrates, such as starch and dietary fibre. Additionally, it has a significant amount of protein (14–16%) of high quality, as it includes all essential amino acids, including lysine and arginine, which are often not present in other plant-based protein sources. Buckwheat is also rich in vitamins, such as B and E, and minerals (see Table 2). Some studies have revealed that the buckwheat seed contains a small amount of phytate, trypsin inhibitors and lectins, which can reduce the digestibility of proteins and cause allergic reactions in some individuals. The high levels of flavonoids present in buckwheat, particularly rutin, have been found to have antioxidant and anti-inflammatory properties, and they also have prebiotic and probiotic benefits [74].

## 5. Current Food Applications of Ancient Grains

**Figure 1:**
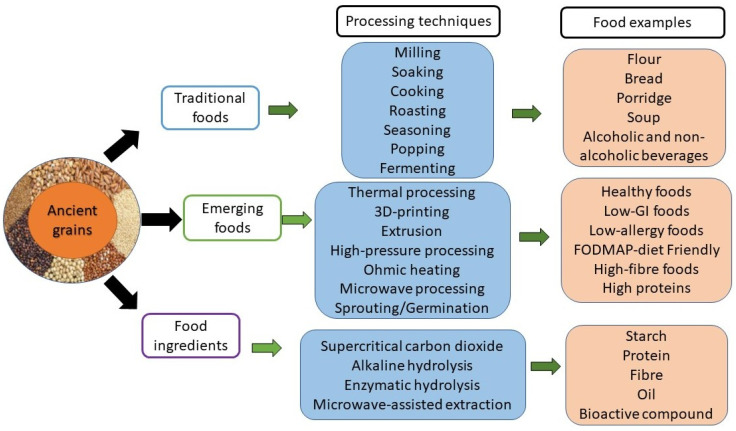
Traditional and emerging processing techniques to convert ancient grains into various products.

Figure 1 provides a summary of some traditional and emerging food-processing techniques of ancient grains. Traditionally, ancient grains have been processed using minimal food-processing techniques to convert them into edible forms with improved organoleptic properties, such as homemade bakery products, porridge, soups, fermented products and ready-to-eat seasoned grains. The common processing methods used for this purpose are de-braning, soaking, roasting, milling, steaming, sprouting, popping and flaking to produce ready-to-eat salted grains and fermented products [22,27,79]. However, with increasing knowledge about the nutritional quality and health benefits of ancient grains, they have been in the spotlight in the production of emerging foods, such as healthy foods, plant proteins, high-fibre foods, low GI foods and allergy-free products, using modern food-processing techniques and often marketed at premium prices. Examples of these modern techniques are extrusion, microwave, ohmic heating, ultrasound, 3D printing and high-pressure processing [11,12,44]. Sprouting/germination and high-pressure processing have been used to reduce the antinutrients and improve the organoleptic properties of the ancient grains, and high-pressure processed ancient grains with reduced antinutrients and improved organoleptic properties have been successfully produced and used in the production of various foods such as pasta and bread [13,15,80]. There is also a growing interest in isolating different functional components from ancient grains, such as starch, protein, bran and fibre, oil and bioactive compounds, which can then be used in the production of healthy foods, nutraceuticals and pharmaceuticals [17,19,20,38]. 

## 6. How Can Ancient Grains Prevent Hunger and Malnutrition?

**Figure 2:**
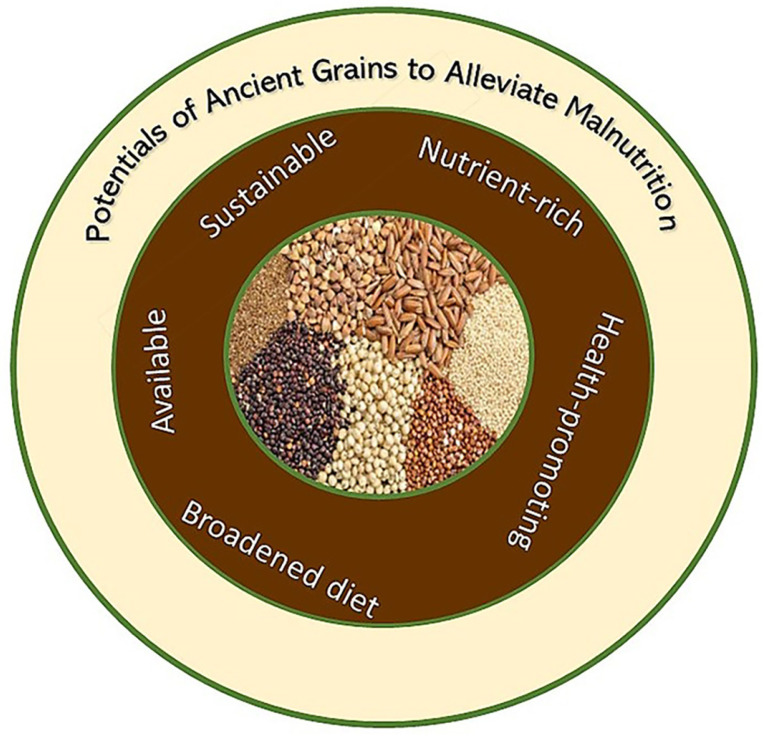
A spectrum of ancient grains illustrating the prospects of ancient grains to fight world hunger and malnutrition.

Ancient grains can have a great contribution in mitigating food hunger and malnutrition for several reasons, as discussed below (summarised in Figure 2).

### 6.1. Ancient Grains as Highly Resilient Crops

Drought, extreme temperatures, water shortage, nutrient-poor soils and uncontrolled plant diseases and pests are the main factors threatening modern grains, causing food shortages and famine, especially in developing countries with a high prevalence of hunger. Unlike modern cereals, ancient grains have a diverse genetic ability to withstand many biotic and abiotic stresses [8]. This feature is highly valuable in supporting food security and establishing resilient agriculture in a wide range of climates [8].

### 6.2. Ancient Grains as Nutrient-Dense and Health-Promoting Foods

Ancient grains are natural and economical sources of nutrients and bioactive compounds that can provide a sufficient amount of carbohydrates, high-quality proteins, essential amino acids, dietary fibres, minerals, vitamins and bioactive compounds to supply energy and nutrients for healthy body functions and to combat hunger and malnutrition [11,25]. Many ancient grains such as sorghum, amaranth and chia seeds are rich sources of protein and lysine which is the lacking amino acid in modern grains. Thus, they can be an excellent source of plant-based protein, which is in high demand, especially in developing countries, due to the high cost of animal products that results in protein deficiency. Ancient grains are also great sources of vitamins (vitamins B1, B3, B6, folate and vitamin E) and minerals, especially Fe, Zn and Ca, and can be used to address minerals and vitamin deficiency caused by hunger and malnutrition. However, due to the presence of some antinutrients, developing pre-treatment technologies are required to increase the digestibility of proteins and the bioavailability of the minerals and vitamins [11,18].

Some tested ancient grains such as oat, teff, and sorghum naturally contain high levels of resistant starch with low digestible starch and hence are considered low GI foods. Resistant starch, which is not digestible in the body, acts as a dietary fibre with numerous health benefits, including appetite reduction and reducing the risk of obesity, improving postprandial glucose and insulin responses and also acting as a prebiotic compound for improving gut microbiome in the human body [18].

Ancient grains have shown positive effects to address many health issues related to malnutrition and hunger, such as cardiovascular diseases, diabetes type 2, cancer, weight control, IBS (irritable bowel syndrome) and digestion. It has also been reported that the consumption of ancient grains could improve both gastrointestinal symptoms (e.g., IBS) and inflammatory profiles in different groups [12,49,81].

### 6.3. Ancient Grains to Diversify Food Sources

Unprecedented environmental, climate and political problems threaten food security by disrupting the food supply chain hence supply and production diversification is of great importance. It is also well known that a diverse diet (i.e., consisting of a larger number of food sources, e.g., a number of cereals and pseudocereals) can provide a wide range of nutrients required for human health and hence planning a diverse diet is an important strategic approach for tackling hunger and malnutrition. Currently, only a few modern grains, including wheat, rice and corn, are the major grain contributors (sources) to human nutrition. However, a food system based on only modern grains is not sustainable due to their high susceptibility to biotic and abiotic stresses. In addition, the selection of high-yielding cultivars reduces their nutritional quality. The inclusion of ancient cereals in our diet can therefore diversify food sources, support food security and enrich the nutritional quality of the foods [5].

### 6.4. Ancient Grains for Special Diet Foods

People who require a special diet, such as those suffering from a digestive disorder, metabolic syndrome, food allergy and intolerance, are more at risk of malnutrition. Most ancient grains can be used as healthy and highly nutritious gluten-free alternatives to modern grains such as wheat, rice and corn [18,34]. Ancient grains cause fewer allergic reactions and are also more tolerable than normal grains for FODMAP diet foods [49].

Some ancient grains are rich sources of resistant and slowly digestible starch, which can be used for the production of low GI and low-calorie foods suitable for weight control and diabetes [13,15]. They can be used as a source of plant protein required in meat-free diets and also in countries where access to other protein sources is limited. Some ancient cereals have been added to produce low-fat foods. For instance, the hydrophilic properties of chia seeds enable them to be substituted for eggs and fat in food recipes [22].

### 6.5. Ancient Grains to Support Small-Scale Farmers

Economic crisis and poverty are directly related to food shortage and hunger. Small-scale farmers are highly vulnerable to job insecurity due to the high cost of modern agriculture. Growing ancient grains in developing countries can create jobs for small-scale farmers and support their income which facilitates access to better nutrition with minimal inputs such as water, land and fertiliser. This can also increase access to locally grown, highly nutritious and affordable food sources and reduce the need for importing grains from other countries [8].

## 7. Major Shortcomings of the Ancient Grains in to Fight against Hunger

Despite many advantages and health benefits of ancient grains, they have remained under-utilised due to their limitations, including low production yields and hence reduced availability compared to modern grains; lack of knowledge and technology of pre- and post-harvest processing; the presence of some anti-nutritional factors such as phytic acids, tannins and lectins (some causing bitter taste); and limited knowledge on their food processing, consumer perceptions, sensory studies and marketing. Since ancient grains have been neglected for many years, limited knowledge is available about their germplasms, different varieties, production, functionality and value-addition [5,18].

## 8. Concluding Remarks

For fighting hunger, relying only on high-yielding modern grains is highly unreliable and can lead to catastrophic outcomes because the existing major crops are highly prone to adverse climate changes and low-input environments and are not nutritionally balanced. Despite having low yields, ancient grains have excellent nutritional profiles and health benefits and are highly resistant to various biotic and abiotic stresses. Thus, growing a balanced combination of both modern and ancient grains is required to obtain more sustainable, diversified and nutritious foods to tackle hunger and malnutrition. Nevertheless, ancient grains are still highly under-utilised, and further research is necessary to turn these valuable grains into a real opportunity to tackle global hunger.

A research priority is to improve the production yields of ancient grains to increase their mass production and economic return, e.g., by selecting and breeding different cultivars and their best production performance conditions. It is also necessary to find feasible, industry-friendly and environmentally safe strategies to eliminate antinutrients, which have negative effects on the nutritional quality, health benefits and sensory properties of ancient grains.

With the fast-pacing food industry, there is an urgent need to use novel technologies to create functional food ingredients that replace existing ingredients in small- and large-scale food production settings. Moreover, underpinning the effects of modern food-processing techniques on the physicochemical, quality, nutritional properties, stability and sensory attributes of ancient grains is of prime importance.

Further research is required to develop new, affordable and healthy foods from ancient grains for special diet requirements.

It is also necessary to identify ancient grains that are naturally low in allergens and antinutrients, such as phytate and tannins, to improve the bioavailability of the nutrients.

## Figures and Tables

**Table 1:** Chemical composition of ancient grains (%, dry basis).

Ancient Grains	Carbohydrate	Starch	Dietary Fibre	Protein	Lipid	Ash	References
Spelt wheat	68–72	52–65	10.7–13.9	14.6–15.7	1.7–1.9	1.7–1.9	[6,7]
Emmer wheat	63.5–68.5	52–65	7.2–12.0	14–16	1.8–2.8	2.1–2.3	[6]
Einkorn wheat	60–64	58–68	9.3–12.8	13.5–15.4	2.0–2.8	2.6–2.2	[6]
Barley	64–75%	59.1–61.6	12.8–17.2	11.7–13.6	1.4–3.9	1.5–4.5	[11]
Oat	75–80	54.9–63.6	8.5–13	10.0–15.0	3.0–8.0	1.7–1.9	[12]
Millet	65–80.6	62–70	1.52–4.65	6.2–14.5	1.2–8.2	0.73–3.3	[13]
Wild rice	71–84	56–79	1.15–1.93	10–15.5	0.7–1.23	1.1–2.0	[14]
Green wheat	73–80	45–68	12.0–19.0	11.0–15.0	1.32–2.7	0.8–2.0	[15]
Sorghum	57–83	55–79	1.0–7.4	7–15	2–3	0.68–4.2	[16,17]
Amaranth	63.8–65.2%	65–75%	6.7–11.4%	12.5–13.5%	5.7–7.2%	1.5–2.8%	[18,19]
Quinoa	65	58.1–64.2	16.5	12.8	3.9	2.4	[20]
Teff	67	-	12.1	13	5	2.2	[21]
Chia	3.4	-	21.1–33.3-	18.9	31.2	2.9	[22]
Buckwheat	65	54.5–57.4	13.8	15.1	2.9	1.9	[23,19]

**Table 2:** Major micronutrients, antioxidants and health benefits of the ancient grains.

Ancient Grains	Vitamins	Minerals	Main Antioxidants	Health Benefits	Ref.
Spelt	Vit. B1: 0.14–0.17 mg/100 g	Zn: 47 mg/kg Fe: 50 mg/kg P: 4.7 g/kg	Ferulic acids: 223–502 µg/g	Modulating postprandial glycemia and insulin level	[10]
Emmer	Vit. B1: 0.42 mg/100 g	Zn: 54 mg/kg Fe: 49 mg/kg P: 5.1 g/kg	Ferulic acids: 323–711 µg/g	Reducing total cholesterol, LDL cholesterol and blood glucose	[24]
Einkorn	Vit. B2: 0.45 mg/100 g	Zn: 36–84 mg/kg Fe: 32–85 mg/ kg Mn: 26–92 g/kg P: 5.2 g/kg Cu: 4.1–10 mg/kg	Ferulic acids: 207–442 µg/g	Enhancing blood carotenoid level, antioxidant activities that reduce cardiovascular disease and hypoallergenic effects	[6,25]
Barley	Vit. B1: 0.35 mg/100 g; Vit. B2: 0.091 mg/100 g Vit. E: 0.85–3.15 mg/100 g	Zn: 6–245 mg/kg Fe: 26–334 mg/kg P: 3320–5020 mg/kg	Ferulic acid: 4.5–102 mg/100 g	Reducing blood cholesterol levels and increasing insulin response in diabetics, lowering blood glucose levels, weight control, gut regulation, preventing colon cancer	[26]
Oats	Vit. B1: 50 mg/kg; Vit. B2: 1.4 mg/kg	Zn: 39 mg/kg Fe: 38 mg/kg P: 3.7 g/kg	Ferulic acids: 24–40.8 µg/100 g	Reducing the serum cholesterol, excellent antioxidant and anti-inflammatory activities, improving gut health and reducing risks of cardiovascular diseases	[27]
Millet	Vit. C: 0.04 mg/100 g Vit. A: 0.015 mg/100 g Vit. B1: 0.15–0.52 mg/100 g Vit. B2: 0.09–0.28 mg/100 g Vit. B3: 1.1–4.5 mg/100 g	Ca: 23–350 mg/100 g Fe: 1.18–53.39 mg/100 g P: 255–509 mg/100 g Zn: 0.73–4.2 mg/100 g Mg: 78–201 mg/100 g	TPC: 36–445 mg/100 g TFC: 51–202 mg/100 g Ferulic acid: 3.3–36.6 mg/100 g	Antioxidative and antiproliferative activities; therapeutic intervention in type 2 diabetes; alleviation of cardiovascular diseases, liver injury and cancer; lowering blood pressure.	[28]
Wild rice	Vit. B1: 0.30–0.63 mg/100 g Vit. B2: 0.07–0.2 mg/100 g Vit. E: 0.2–4.8 mg/100 g	Ca: 21–24 mg/100 g Fe: 1.60–3.17 mg/100 g Mg: 106–120 mg/100 g Mn: 0.93–1.45 mg/100 g P: 236–384 mg/100 g K: 145–244 mg/100 g Na: 1.34–5.86 mg/100 g Zn: 1.25–2.83 mg/100 g	TPC: 16.98–58.8 mg/100 g Ferulic acid: 24.1–35.5 mg/100 g Sinapic acid: 5.5–9.6 mg/100 g p-coumaric acid: 1.1–4.3 mg/100 g	Alleviation of insulin resistance and lipotoxicity; atherosclerosis prevention; anti-inflammatory, anti-hypertensive and immunomodulatory effects; antiobesity; antianaphylactic actions; prevention and treatment of cardiovascular disease; cholesterol-lowering and anti-atherogenic effects	[29,14]
Green wheat	Vit. B: 1.80 mg/100 g Vit. B2: 0.19 mg/100 g Vit. B3: 1.30 mg/100 g Vit. C: 4.5 mg/100 g Vit. E: 0.2–0.6 mg/100 g	Na: 4–12.5 mg/100 g Ca: 32–63 mg/100 g K: 369–451 mg/100 g Mg: 160–202 mg/100 g P:412 mg/100 g Cu: 0.49 mg/100 g	Ferulic acid: 1444 mg/100 g	Preventive and treatment effects on chronic degenerative diseases caused by oxidative stress; reducing the risk factors for obesity, diabetes, cardiovascular diseases and cancer; antianemia effects	[14,29,30]
Sorghum	Vit E: 1.95 mg/100 g α-tochopherol: 0.122–0.525 mg/100 g Vit A (β-carotene): 0.054–0.134 mg/100 g Thiamine: 0.08 mg/100 g Riboflavin: 0.21 mg/100 g Pyridoxine: 0.17 mg/100 g	Ca: 665.6 mg/100 g Fe: 168.8 mg/100 g K: 26,940 mg/100 g Mn: 141.2 mg/100 g Na: 292.5 mg/100 g P: 32,727 mg/100 g Zn: 432.8 mg/100 g Mg: 12,010 mg/100 g	TPC: 109–1040 mg/100 g TFC: 11–61 mg/100 g Ferulic acid: 2.40–86.8 mg/100 g caffeic acid: 1.43–8.17 mg/100 g p-coumaric acid: 0.68–8.17 mg/100 g	Reducing the risk of cardiovascular disease, cancer, diabetes, dyslipidaemia and coeliac disease; antiallergic properties	[16,31,32,33,34,35,36,37,38,39,40]
Amaranth	Vit. B3: 64.4 mg/100 g Vit. E: 1.54 mg/100 g Vit. C: 64.4 mg/100 g	Fe: 7.61 mg/100 g Zn: 287 mg/100 g Mg: 248 mg/100 g Mn: 3.3 mg/100 g P: 508 mg/100 g Cal: 159 mg/100 g	Protocatechuic *p*-Hydroxybenzoic *p*-coumaric Ferulic acid	Anti-radical Antioxidant Anti-inflammatory Anti-diabetic Anti-cancer Improving gut health	[23,41]
Quinoa	Vit. B3: 0.01–8 mg/100 g Vit. E: 24.7 mg/100 g Vit. C: 4–49.3 mg/100 g Folate: 0.2 mg/100 g	Fe: 5.5 mg/100 g Zn: 1.8 mg/100 g Mg: 206 mg/100 g Cal: 32.9 mg/100 g	Gallic acid Caffeic acid Ferulic acid p-coumaric p-Hydroxybenzoic acid Vanillic acid	Antioxidant activity Anti-obesity Antimicrobial Skin protection Anti-inflammatory Anti-diabetic Preventing cardiovascular disease and childhood malnutrition Improving gut health	[23,41]
Teff	Vit. B1: 0.3 mg/100 g Vit. B3: 3.3 mg/100 g Vit. E: 0.08 mg/100 g Vit. C: 88 mg/100 g	Fe: 7.63 mg/100 g Zn: 3.63 mg/100 g Mg: 184 mg/100 g P: 427 mg/100 g K: 427 mg/100 g Cal: 180 mg/100 g	Catechin Ferulic acid Rosmarinic acid *p*-coumaric acid	Anti-radical Antioxidant Anti-inflammatory	[42]
Chia	Vit. B2: 0.17 mg/100 g Vit. B3: 8.83 mg/100 g Vit. B1: 0.62 mg/100 g Vit. E: 8.1 mg/100 g Vit. C: 1.6 mg/100 g	Ca: 455 mg/100 g P: 585 mg/100 g K: 585 mg/100 g MG: 340 mg/100 g Fe: 8.54 mg/100 g Zn: 3.7 mg/100 g	Caffeic acid Chlorogenic acid Quercetin Kaempferol	Anti-hypertensive Antioxidant activity Anticholesterolemic Anthropometrics Hypoglycemic	[43,22]
Buckwheat	Vit. B3: 2.1–18 mg/100 g Vit. E: 9.5–16.4 mg/100 g	Fe: 4.7 mg/100 g Zn: 1.0 mg/100 g Mg: 203 mg/100 g Ca: 60.9 mg/100 g	Rutin Ferulic acid caffeic acid gallic acid *p*-Coumaric	Anti-inflammatory Anti-hypertensive Antioxidant activity Anti-obesity Antidiabetic activity Anti-cancer Improving gut health	[41,44]

## Data Availability

The data presented in this study are available on request from the corresponding author.
